# Spatiotemporal Heterogeneity of Zika Virus Transmission in Indonesia: Serosurveillance Data from a Pediatric Population

**DOI:** 10.4269/ajtmh.21-0010

**Published:** 2021-05-03

**Authors:** R. Tedjo Sasmono, Edison Johar, Benediktus Yohan, Chairin Nisa Ma’roef, Paul Pronyk, Sri Rezeki Hadinegoro, Elizabeth Jane Soepardi, Alain Bouckenooghe, William A. Hawley, Ronald Rosenberg, Ann M. Powers, Amin Soebandrio, Khin Saw Aye Myint

**Affiliations:** 1 Eijkman Institute for Molecular Biology, Jakarta, Indonesia;; 2 UNICEF Indonesia, Jakarta, Indonesia;; 3 Faculty of Medicine and Cipto Mangunkusumo Hospital, Universitas Indonesia, Jakarta, Indonesia;; 4 Ministry of Health of the Republic of Indonesia, Jakarta, Indonesia;; 5 Sanofi Pasteur, Lyon, Rhône-Alpes, France;; 6 Centers for Disease Control and Prevention, Atlanta, Georgia;; 7 Centers for Disease Control and Prevention, Fort Collins, Colorado

## Abstract

The presence of Zika virus (ZIKV) in Indonesia has been recognized since the 1970s, but its transmission dynamics there have been poorly understood. To understand more fully the geographic distribution and burden of ZIKV infection, we performed retrospective serological tests on specimens collected from asymptomatic children age 5 to 9 years old living at 30 sites in 14 provinces. Of 870 serum samples tested, 9.2% were found to be positive for anti-ZIKV antibodies, as confirmed by plaque reduction neutralization assays. This was the same overall prevalence reported previously for 1- to 4-year-old children collected at the same sites at the same time. Together with geographic differences in seroprevalence between the age groups, these data suggest that, although ZIKV might be endemic in Indonesia, its occurrence has been focal and episodic.

The global epidemic of Zika virus (ZIKV) began in Micronesia in 2007 and peaked in the tropical western hemisphere, where it had not been found previously, from 2015 to 2016.^1^ Transmitted predominately by *Aedes aegypti*, ZIKV is rare among flaviviruses for causing birth defects and for being capable of sexual transmission.^1^ No vaccine is yet available to protect against ZIKV, and the post-epidemic threat it presents now throughout the tropics is poorly understood.

Evidence of ZIKV transmission has been reported from Asia for decades.^1^ The first cases from Indonesia, based on serological evidence, were reported from Java in 1981, and active transmission was confirmed molecularly from Sumatra in 2015.^2,3^ The dengue viruses (DENV) and chikungunya virus (CHIKV), also transmitted by *Ae. aegypti*, occur widely in Indonesia, so there is reason to believe that, despite infrequent reports of ZIKV as a cause of disease, it has long been endemic in the country. ZIKV clinical symptoms are typically mild and are likely often overlooked or misdiagnosed as DENV, a problem compounded by a high level of serological diagnostic cross-reactivity among the flaviviruses.^4^

There have been few reported seroprevalence studies on ZIKV. In a review of data from 20 countries, the majority of studies did not confirm antibody results with virus-specific neutralization assays.^5^ We recently reported the presence of ZIKV antibodies in blood collected during a DENV cross-sectional serosurvey in 2014 from healthy 1- to 4-year-old Indonesian children, as confirmed by the plaque reduction neutralization test (PRNT_90_).^6^ In this report we analyze antibody levels in sera collected in the same 2014 serosurvey, but from older children.^7^ This group includes 5- to 9-year-old children (range, 60–119 months) born between November 2004 and November 2009; the 1- to 4-year-olds (range, 12–59 months) were born November 2009 to November 2014.

A total of 870 serum samples from healthy 5- to 9-year-old children, which had been collected from 30 districts in 14 provinces during October and November 2014, were tested.^7^ ZIKV-specific PRNT_90_ were performed using a method adapted from a previously published protocol.^6^ The virus used was ZIKV strain JMB-185, isolated from a febrile case from Jambi, Sumatra.^3^ Briefly, sera were screened initially for ZIKV antibodies by PRNT_90_ at a 1:10 serum dilution. Positive samples were subjected to ZIKV and DENV PRNT_90_ combo tests in which sera were tested against ZIKV and DENV-1, -2, -3, and -4. Samples were considered to be true ZIKV seropositives when positive for anti-ZIKV antibodies and negative for anti-DENV antibodies, or when the ZIKV PRNT_90_ titer was ≥4-fold higher than all DENV PRNT_90_ titers. Specimens were categorized as flavivirus seropositive only when anti-ZIKV antibodies were present, but at titers of < 4-fold higher than any anti-DENV antibodies.

During the initial screening of 870 sera, we detected possible ZIKV-positive antibodies in 150 (17.2%) specimens. When tested further with the combo ZIKV–DENV PRNT_90_, 80 (53.3%) specimens were determined to be true ZIKV seropositives. Of the remaining 70 flavivirus-positive specimens, 28 (18.6%) were identified as true DENV seropositives and 42 (28.0%) remained indeterminate, with both ZIKV and DENV antibodies detected. Overall, 9.2% (95% CI, 8.2–10.2) of the 5- to 9-year-olds were positive for the presence of anti-ZIKV antibodies (Table 1).

**Table 1:** Results of 90% plaque reduction neutralization tests on sera testing positive for Zika virus from 5- to 9-year-old children living in 14 provinces of Indonesia.

Province	Sample size (*N*)	ZIKV and DENV PRNT_90_ combo test			
		Initial ZIKV positive*	Confirmed ZIKV positive†	Confirmed DENV positive	Indeterminate flavivirus positive
Aceh	29	2	0	1	1
North Sumatera	29	3	3	0	0
West Sumatera	29	4	1	1	2
Jambi	29	6	3	2	1
Lampung	29	0	0	0	0
Banten	58	4	4	0	0
DKI Jakarta	87	18	10	3	5
West Java	203	31	16	6	9
Central Java	116	37	21	4	12
East Java	145	28	12	9	7
Bali	29	4	2	0	2
East Kalimantan	29	3	2	0	1
South Sulawesi	29	0	0	0	0
Southeast Sulawesi	29	10	6	2	2
Total	870	150	80	28	42

DENV = Dengue viruses; PRNT_90_ = plaque reduction neutralization test; ZIKV = Zika virus.

* Serum samples that neutralize 90% ZIKV challenges at 1:10 serum dilution on initial ZIKV PRNT_90_ screening.

† Serum samples that neutralize ZIKV only or ≥ 4-fold ZIKV PRNT_90_ titer compared with any DENV PRNT_90_ titer.

Dengue seroprevalence in these children increased with age.^7^ The site-specific degree of difference between the 1- to 4-year and 5- to 9-year groups varied, but in each the prevalence in older children was greater (Figure 1), indicating a perennial risk for infection with one or more of the four DENV, a pattern well established in Southeast Asia. By contrast, the overall seroprevalence of ZIKV in the 5- to 9-year-old group was the same as that found in the 1- to 4-year-old group (9.1%).^6^ There was approximate equality in prevalence between the age cohorts at each of the five sites on the island of Java (Banten, Jakarta, West Java, Central Java, East Java), where the greatest number of specimens was collected (Figure 2). When Java is treated as a whole, by summing for the five sites, the proportion of seropositives among the 1- to 4-year-olds (0.0104, 48 of 463) and among the 5- to 9-year-olds (0.0103, 63 of 609) were identical (score 95% CI for the difference = 0.000223; 95% CI, –0.0362 to 0.0382). This suggests that, in Java, exposure occurred predominately during 2009 through 2014. Elsewhere, there were apparent local differences in seroprevalence, but none of these site-specific differences were statistically significant (Fisher’s exact *P* test); most sites were represented by no more than 30 specimens per age group. At two sites (Aceh and South Sulawesi), there was no evidence of ZIKV exposure during the entire period (2004–2014), whereas in Bali and Southeast Sulawesi, substantially greater prevalence in the older cohort could indicate most transmission occurred during 2004 through 2009. There is no obvious explanation to explain why prevalence appears limited to the younger populations in West Sumatra, Jambi, and Lampung, except as a consequence of small sample size.

**Figure 1.**
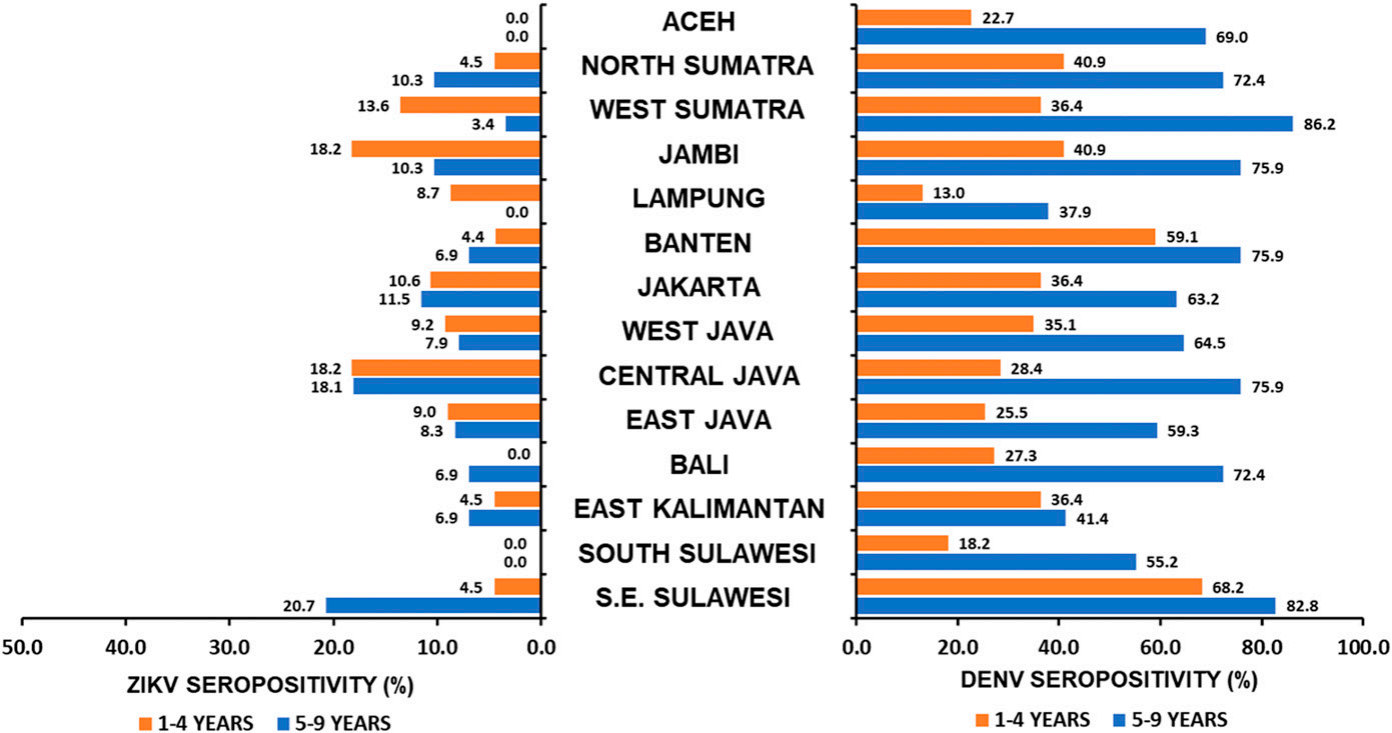
Summary of Zika virus (ZIKV) and Dengue viruses (DENV) seroprevalences in children age 1 to 4 years old and 5 to 9 years old in Indonesia by province. ZIKV seroprevalences for 1- to 4-year-olds^6^ and DENV seroprevalences for both age groups^7^ are from previously reported studies.

**Figure 2.**
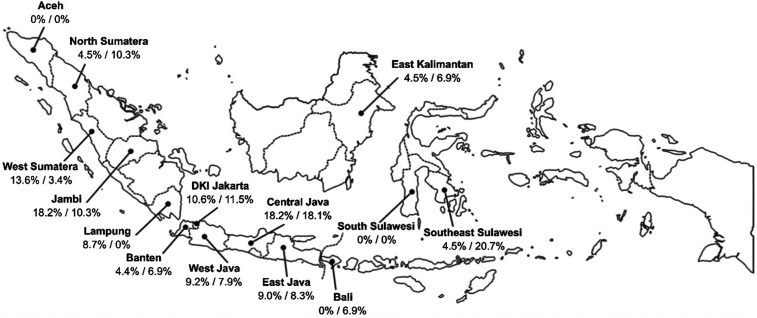
Geographic distribution of Zika virus (ZIKV) seropositive children, by province, Indonesia, October through November 2014. Labels indicate the percentages of 1- to 4-year-old^6^ and 5- to 9-year-old children who tested as true ZIKV seropositive (serum samples that neutralize ZIKV only or ≥ 4-fold ZIKV plaque reduction neutralization test [PRNT_90_] titer compared with any dengue viruses PRNT_90_ titer).

It is possible that ZIKV is endemic in Indonesia, which comprises about 6,000 inhabited islands spread across nearly 2 million km^2^, but our results suggest geographically specific epidemic transmission. Although ZIKV infection is caused by a single virus species, which presumably elicits long-lasting immunity, tests for DENV antibodies typically measure nfection with any of the four related DENV, each of which produces life-long immunity only to itself. A more revealing comparison with ZIKV of site-specific, longitudinal arbovirus transmission might be with the monospecific CHIKV, an alphavirus also transmitted by *Ae. aegypti*. Although we cannot find an equivalent study of CHIKV seroprevalence in Indonesia, there are several describing an unpredictable pattern of epidemics.^8^

The history of ZIKV transmission during the 70 years preceding the Yap epidemic is murky. Although it was first described from Uganda, its origin and first appearance in Southeast Asia are unknown. Nevertheless, it seems reasonable to surmise that it might have circulated within Indonesia well before its discovery there in 1977. It is also likely that ZIKV has been reintroduced periodically from elsewhere in Asia. Early phylogenetic research shows clear linkages between Indonesian specimens and those from Thailand, Myanmar, Cambodia, and the Philippines.^9–14^

This study has limitations. ZIKV and DENV share a high degree of homology in the E protein, a major target for neutralizing antibodies.^15^ Cross-reactivity between DENV and ZIKV restricted our confirmatory neutralization studies to subjects younger than 10 years old; beyond this age, the high DENV prevalence in Indonesia would make discrimination of ZIKV increasingly uncertain. Japanese encephalitis virus (JEV) also occurs in Indonesia, but much less commonly than DENV. None of the 1- to 4-year-old cohort was positive for JEV, and we did not test for it in the 5- to 9-year-old group.^6^ The 28% positive specimens classed as indeterminate contain an unknown proportion of ZIKV exposures. The small sample sizes from most sites limited our ability to analyze results statistically. In the largest samples, from Java, there were no statistically significant differences between age groups. Last, these data shed no light on the possibility of protective cross-immunity between DENV and ZIKV, which might limit incidence.

This study is one of the few reported widescale uses of antibody prevalence to examine the history of ZIKV transmission. By comparing the infection histories of two pediatric age cohorts, we conclude that despite the lack of clinical evidence, ZIKV is endemic in Indonesia or is reintroduced periodically, and is apparently characterized by geographically distinct, self-limiting outbreaks. The lack thus far of evidence for ZIKV as a cause of congenital defects in Indonesia might be a result, in part, of many adults having been immunized naturally as children.^16^ A fuller understanding of the risk and burden of ZIKV is necessary everywhere it occurs.
